# Case report: A novel FBXW7 gene variant causes global developmental delay

**DOI:** 10.3389/fgene.2024.1436462

**Published:** 2024-09-19

**Authors:** Yu Wang, Xiaoping Ma, Hua Li, Yanrui Dai, Xiaochen Wang, Li Liu

**Affiliations:** ^1^ College of Clinical Medicine, Ningxia Medical University, Yinchuan, China; ^2^ Department of Pediatric Rehabilitation, The First People’s Hospital of Yinchuan, Yinchuan, China

**Keywords:** *FBXW7*, hypertonia, global developmental delay, intellectual disability, trio-WES

## Abstract

**Objective:** To investigate a case of neurodevelopmental disorder caused by mutation of *FBXW7*.

**Methods:** Clinical data were collected from the patient, trio-WES (whole-exome sequencing) was performed on the patient and his parents (trio), and the results were verified by Sanger sequencing. RESULTS: The patient was a 2-year and 1-month old male who presented with facial dysmorphism (prominent forehead, ocular hypertelorism, and low nasal bridge), global developmental delay, language impairment, hypertonia, labial hemangioma, hydrocele, and overgrowth. The trio-WES confirmed that the child had a pathogenic *de novo FBXW7* gene variant, c.1612C>T (p.G1n538*), a heretofore unreported locus.

**Conclusion:** This case of developmental delay, hypotonia, and impaired language (OMIM: #620012) related to a mutation in *FBXW7*, is a rare genetic disorder, newly identified in recent years, and seldom reported. The presence of hypertonia, labial hemangioma, and hydrocele in this child suggests significant phenotypic heterogeneity of the disease, and the discovery of new mutant loci enriches the spectrum of pathogenic variants of the disease.

## 1 Introduction

Developmental delay, hypotonia, and impaired language (DEDHIL, OMIM: #620012) is a rare autosomal dominant disorder caused by heterozygous variants in the FBXW7 gene, which has been shown to regulate cell cycle progression, cell signaling, and development via the ubiquitin proteasome system (UPS) targeting cyclinE1/2, Notch, c-Jun and other substrates for degradation (Stephenson et al., 2022; De La Cova, 2023). The functionality of FBXW7 in brain development is mediated by Notch and c-Jun. The Notch signaling pathway acts through the process of lateral inhibition and plays an important role in neuronal and glial differentiation (Snyder et al., 2012). Another important regulator of neuronal viability is c-Jun, and FBXW7 can mediate the expression of c-Jun protein through excitotoxicity in response to glutamate-induced hydrolysis to attenuate neuronal apoptosis (Yang et al., 2021). Thus, mutations in FBXW7 can lead to neurodevelopmental disorders (Stephenson et al., 2022). Stephenson et al. (2022) studied 35 patients with DEDHIL from around the world and identified the main clinical features of the disease as neurodevelopmental abnormalities (34/35, 97.1%), including mild to moderate (27/35, 77.1%) neurodevelopmental disorder (NDD) or intellectual disability (ID), which was severe in a few patients (3/35, 8.6%), and organic brain changes in 13 of the 17 patients who underwent neuroimaging examinations (76.5%). Notably, hypotonia is one of the key features for the clinical diagnosis of DEDHIL, occurring in 62.9% of reported patients (22/35) (Stephenson et al., 2022). In addition, there is clinical heterogeneity in the facial dysmorphism and neurologic appearance patterns of DEDHIL (Stephenson et al., 2022). Currently, 35 patients with DEDHIL have been reported. Most of them were male (M:F = 26:9) and only one suspected East Asian patient (female) has been reported (Stephenson et al., 2022), which is an epidemiologic feature that has not yet been explained and needs in-depth study.

In this paper, we report the first case of early childhood DEDHIL in a male in China. In addition to the phenomena of global developmental delay (GDD), neurodevelopmental disorder (NDD), and overgrowth, there was a combination of hypertonia, hydrocele, and labial hemangioma, which is the first time that these were found among the clinical manifestations of an identified case of DEDHIL. While the overgrowth phenomenon has only been reported in one case in the past, further clinical validation has demonstrated that FBXW7 plays an important role in inhibiting cell growth (Yang et al., 2021).

## 2 Case description

The male child’s age was 2 years and 1 month. The mother had two pregnancies with one normal delivery (G2P1). The prenatal examination found no abnormality. The mother denied a history of adverse pregnancy. During the neonatal period at 3 days after birth, the child developed a fever (38.3°C–38.8°C) and was transferred to the neonatology department of the hospital. During hospitalization, on the 3rd day after birth, liver function tests showed serum total bilirubin 326.9 μmol/L and indirect bilirubin 325.9 μmol/L. Waist cerebrospinal fluid puncture indicated intracranial infection, hemoglobin 101 g/L. On the 6th day after birth, he was given a cranial CT (computerized tomography) scan, which showed hemorrhage in the lateral ventricles on both sides, a subarachnoid hemorrhage, and diffuse reduction of cerebral white matter density on both sides of the brain, neonatal ischemic-hypoxic encephalopathy was not excluded.

A cranial MRI (magnetic resonance imaging) showed a small amount of blood in the lateral ventricles (bilaterally), a small amount of subarachnoid hemorrhage (late subacute stage), and mild hydrocephalus, also an MRA (magnetic resonance angiography) and MRV (magnetic resonance venography) showed no abnormality. The final diagnosis was neonatal intracranial hemorrhage, neonatal intracranial infection, neonatal hyperbilirubinemia, hemangioma (upper lip), and mild anemia. He was treated with anti-infective therapy (meropenem for 7 days and ceftriaxone for 7 days) and blue light therapy for jaundice. The total treatment time was 16 days.

**FIGURE 1:**
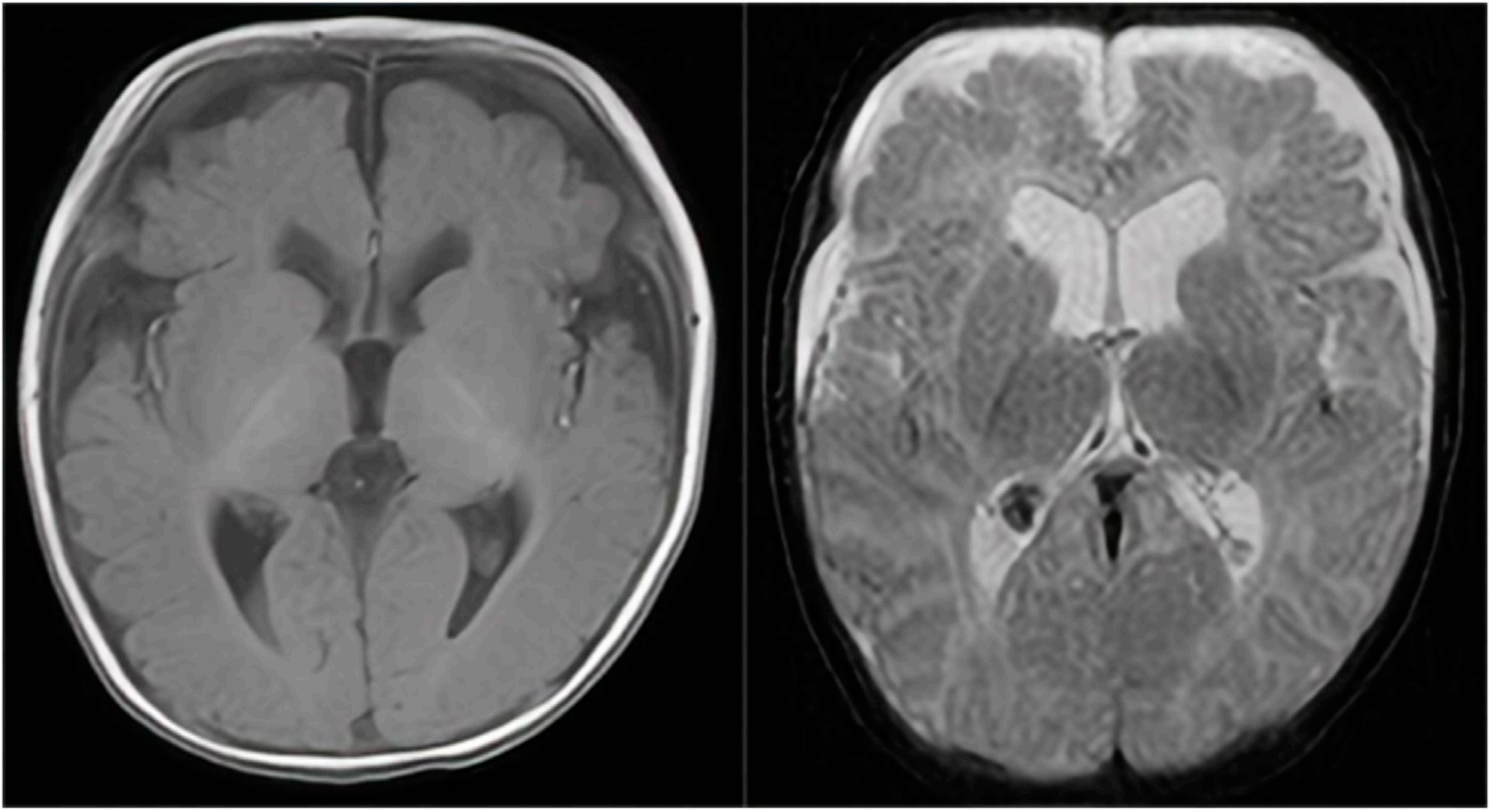
A cranial MRI showed right ventricular hematoma (chronic stage), mild hydrocephalus, and bilateral frontal subdural effusions. The right ventricular hematoma had been absorbed and shrunken compared with the previous one, and ferritin-containing hematomas had been deposited.

With regard to growth and development, the child had a routine outpatient physical examination more than 40 days after birth, and was found to have hypertonia. Subsequent review of the cranial MRI (on the 54th postnatal day) (Figure 1) showed right ventricular hematoma (chronic stage), mild hydrocephalus, and bilateral frontal subdural effusions. The right ventricular hematoma had been absorbed and shrunken compared with the 10th postnatal day one, and ferritin-containing hematomas had been deposited. Audiometry at 3 months indicated normal hearing in both ears. Scrotal ultrasound revealed right testicular hydrocele. He was given intermittent physical therapy by his parents. When he was 4 months, we gave an examination, he was found to have a hydrocele, hypertonia, low muscle strength, and inability to lift his head or roll over after birth. He underwent comprehensive rehabilitation therapy, including physical therapy, occupational therapy, cognitive training, language training, and sensory integration training for more than 6 months and his gross motor skills improved. He could lift his head at 5 months, roll over at 7 months, sit alone at 8 months, and walk alone at 1 year and 5 months, but his stability and coordination were poor. At 1 year and 3 months old, the child could unconsciously call dad or mom.

A physical examination was performed at 2-years and 1-month old. His weight was 18 kg (>3SD), length was 98 cm (>3SD), and head circumference was 50 cm (1SD-2SD). His reactions were slow. He had facial dysmorphism (prominent forehead, ocular hypertelorism, and low nasal bridge). In the middle of the upper lip there was a hemangioma of about 1.5 cm × 1.5 cm which was as high as the skin and also had a clear border. When the hemangioma was pressed, its color faded and the volume was lessened. Language expression was limited to consciously call dad or mom, and wave hello and goodbye. He could not recognize size or color. The child could hold a spoon to eat, but spilled a lot. He could not put on and take off a coat or pants, but could indicate a need for urination and defecation. The muscle tone of the extremities was improved from before, but muscle strength was low with poor balance and coordination. The Gesell Developmental Diagnostic test at 1 year and 10 months yielded a developmental quotient (DQ) of 62.7 (mild developmental delay) (Table1). The score on the Social Life Scale for Infants-Junior High School Students was borderline (9 points). Speech and language delay assessment at 2 year and 1 month showed language delay. With regard to language expression, he could occasionally say individual words. His language comprehension and expression skills were significantly behind his actual age.

**TABLE 1:** Comparison of the Gessell developmental diagnostic assessment scale of the patient at 1 year and 10 months and 2 years and 5 months.

Items (DQ) Age	Gross motor movements	Fine motor movements	Adaptability	Language	Personal & Social competence
Age					
1-year and 10-month	68.4	72.9	60.9	43.4	68.4
2-year and 5-month	80.6	61.5	71.6	51.8	66.9

Considering that the child had facial dysmorphism (prominent forehead, ocular hypertelorism, and low nasal bridge), global developmental delay, language impairment, hypertonia, labial hemangioma, hydrocele and overgrowth, the presence of a hereditary disease couldn’t be excluded. After obtaining the consent of the child’s guardian who signed the informed consent form, 2–3 mL samples of venous blood were drawn from the child and his parents and sent to a genetic testing company for trio-WES.

## 3 Results

**FIGURE 2:**
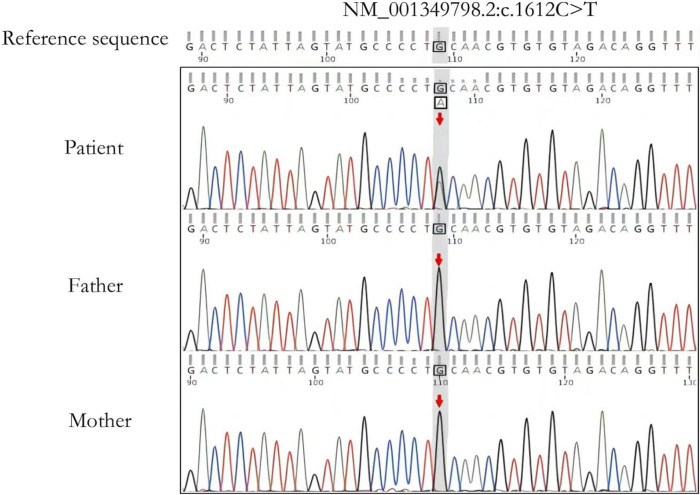
Sanger sequencing validation analysis of the affected child and parents. The child has a *de novo* heterozygous variant c.1612G>A (p.Gln538*) in the *FBXW7* gene. The arrow shows the locus where the variant is located.

Trio-WES detected a *de novo* pathogenic variant in the patient’s *FBXW7* gene (NM_001349798.2: c.1612C>T: (p.Gln538*). This variant was ACMG graded as pathogenic (PVS1+PM2+PP3) and validated by Sanger sequencing (Figure 2). This variant has not been documented by case reports and disease variant databases such as SNP or ClinVar.

## 4 Outcome and follow-up

Currently, there is no specific pharmacologic treatment for DEDHIL. This patient was intermittently given comprehensive rehabilitation in our hospital. We had already followed up for 2 years and 5 months, and the patient’s gross motor skills, adaptability and language comprehension had improved more than before, but social ability and fine motor skills showed no improvement. The child’s language expression was predominantly of the body-language or gestural-language type, and there was no significant progress in verbal expression. The Gesell Developmental Diagnostic test at 2 years and 5 months yielded a DQ of 66.5 (mild developmental delay). Table 1 shows a comparison of neuropsychological developmental evaluations between the two ages.

## 5 Discussion

Developmental delay, hypotonia, and impaired language due to mutations in the FBXW7 gene (DEDHIL, OMIM: #620012) is a recently identified rare autosomal dominant disorder with a common clinical phenotype of generalized developmental delay, varying degrees of intellectual disability, hypotonia, language development delay, and other characteristics including macrocephaly, facial dysmorphism (prominent forehead, ocular hypertelorism, low nasal bridge, mid-facial retraction, high palatal arches, etc.), congenital heart disease, feeding difficulties, constipation, cryptorchidism, epileptic seizures, abnormalities in brain imaging, abnormalities of the corpus callosum, delayed development of the myelin sheath, and cerebellar atrophy (Stephenson et al., 2022). The onset of DEDHIL is most often in infancy, with some individual variability in phenotype and severity, and most patients have a *de novo* variant, with a small percentage of patients inheriting it from a parent with a mild phenotype.

Our diagnosis of DEDHIL was supported by the presence of major clinical phenotypes and the results of genetic tests on this child; yet, the differences in his phenotype from previously reported cases reflect the phenotypic heterogeneity of DEDHIL. Physical examination of the child more than 40 days after birth revealed hypertonia, which differs from the previously known features of DEDHIL. In addition, hemangioma and hydrocele have not been reported in previous cases. Although the patient had a cerebral hemorrhage and an intracranial infection during the neonatal period, a non-specific phenotype due to non-genetic factors cannot be excluded, and proving the association of hypertonia with DEDHIL will depend on the discovery of more cases like this one.

A particularly interesting fact is that the child exhibited intellectual disability combined with hemangioma due to mutation in the FBXW7 gene, which heretofore has not been reported. The exact pathogenesis of infantile hemangiomas is unknown; however, several studies have identified FBXW7 as a positive angiogenesis regulator that counteracts Notch activity in the vascular endothelium (Gu et al., 2022). The Notch signaling pathway may be a novel regulator of the VEGF signaling pathway in infantile hemangiomas. Inhibition of Notch signaling increases the density of blood vessels on the superficial plexus and may lead to excessive neovascularization (Gu et al., 2022). Therefore, FBXW7 mutations may play an important role in the formation of hemangiomas. The child had a history of postnatal cerebral hemorrhage with no apparent cause. Since the FBXW7 plays an important role in angiogenesis, an association of postnatal cerebral hemorrhage with mutations in this gene cannot be ruled out.

FBXW7 is a tumor suppressor, and mutations in this gene can lead to a variety of cancers such as leukemia, breast cancer, gastric cancer, colon cancer, and nephroblastoma (Yeh et al., 2018). Four patients with nephroblastoma were found to have a somatic mutation in the FBXW7 gene, but these patients did not show the phenotype of neurodevelopmental disorders such as developmental delay and intellectual disability (Stephenson et al., 2022). Mutations in this gene have been reported more frequently in tumorigenesis, and only rarely when the tumor is combined with neurodevelopmental disorders. In 2024, Meier-Abt et al. (Meier-Abt et al., 2024) first reported a case of nephroblastoma combined with developmental delay. The child was a male, who was operated on for congenital diaphragmatic hernia, intestinal malrotation, cryptorchidism, and inguinal hernia. He had been lagging behind his peers in gross motor skills, language, and cognitive development since childhood, with mild intellectual disability and hypotonia. The diagnosis of nephroblastoma was confirmed at the age of 7 years, and the trio-WES suggested that the child had somatic mosaicism of the *FBXW7* gene (Meier-Abt et al., 2024). At the same time, the authors demonstrated that the identified variant resulted in downregulation of *FBXW7* and loss of function (Meier-Abt et al., 2024), which provided empirical evidence that patients with DEDHIL may have increased tumor susceptibility. At the time of the 2-year and 5-month follow-up, we have not observed any associated cancers, and long-term follow-up will be required to determine whether there is any increase cancer susceptibility.

In our case, the child presented with global developmental delay (especially language expression delay), facial dysmorphism, hypertonia, low muscle strength, labial hemangioma, overgrowth, hydrocele, and history of cerebral hemorrhage at birth, without hypotonia or gastrointestinal-related symptoms. Among them, hypertonia, lip hemangioma, and hydrocele were the first to be found in previous DEDHIL cases.

In addition, our subject showed overgrowth, and at the age of 2 years and 1 month, his weight was 18 kg (>3SD) and length was 98 cm (>3SD). This was much higher than that of children of the same age, and in the past, only one patient with this type of disease had been reported to have overgrowth (Yang et al., 2021). As FBXW7 plays an important role in the inhibition of cell growth, it was hypothesized that the functional defects due to variants in this gene might be associated with overgrowth (Helbling et al., 2023).

In conclusion, this paper reports a rare case of DEDHIL with phenotypic features such as hypertonia, labial hemangioma, hydrocele, and overgrowth that implies heterogeneity of FBXW7 gene-associated disorders and provides an example of a clinical diagnosis for specialists. In addition, the discovery of the new variant (p.G1n538*) extends the spectrum of pathogenic variants in DEDHIL. Future clinical and functional studies need to elucidate the underlying pathophysiological mechanisms that lead to the observed phenotype, particularly in hypertonia, hemangiomas, overgrowth, etc.

## Data Availability

The original contributions presented in the study are included in the article/supplementary material, further inquiries can be directed to the corresponding author.
